# The Permissive Safe Angle of the Tibial Tunnel in Transtibial Posterior Cruciate Ligament Reconstruction: A Three‐Dimensional Simulation Study

**DOI:** 10.1111/os.13266

**Published:** 2022-04-27

**Authors:** Yuanjun Teng, Gengxin Jia, Lijun Da, Bo Peng, Zhongcheng Liu, Hua Han, Meng Wu, Yayi Xia

**Affiliations:** ^1^ Department of Orthopaedics Lanzhou University Second Hospital Lanzhou PR China; ^2^ Orthopaedics Key Laboratory of Gansu Province Lanzhou University Second Hospital, Lanzhou University Lanzhou PR China; ^3^ Department of Oncology Lanzhou University Second Hospital, Lanzhou University Lanzhou PR China

**Keywords:** Safe angle, Three‐dimensional knee model, Tibial tunnel approach, Transtibial posterior cruciate ligament reconstruction

## Abstract

**Objective:**

To determine the permissive safe angle (PSA) of the tibial tunnel in transtibial posterior cruciate ligament (PCL) reconstruction based on a three‐dimensional (3D) simulation study.

**Methods:**

This was a computer simulation study of transtibial PCL reconstruction using 3D knee models. CT images of 90 normal knee joints from 2017 to 2020 were collected in this study, and 3D knee models were established based on CT data. The tunnel approaches were subdivided into the anterior 1/3 of the anteromedial tibia (T1), middle 1/2 of the anteromedial tibia (T2), the tibial crest (T3), anterior 1/3 of the anterolateral tibia (T4), middle 1/2 of the anterolateral tibia (T5). Five tibial tunnels (T1–T5) were simulated on the 3D knee models. The PSAs, in different tibial tunnel approaches were measured, and subgroup analyses of sex, age and height were also carried out.

**Results:**

The mean PSAs of the tibial tunnels with 5 different approaches (T1–T5) were 58.49° ± 6.82°, 61.14° ± 6.69°, 56.12° ± 7.53°, 52.01° ± 8.89° and 49.90° ± 10.53°, respectively. The differences of the mean PSAs between the anteromedial and anterolateral approaches were significant (*P* < 0.05). However, there was no significant difference of the mean PSA value between the two anteromedial tibial tunnel approaches (T1–T2) (*P* > 0.05), as well as between the two anterolateral tibial tunnel approaches (T4–T5). The patient's anthropomorphic characteristics of sex, age, and height were not associated with the PSAs.

**Conclusions:**

The PSA varied with the anteromedial, tibial crest and anterolateral approaches for transtibial PCL reconstruction, and surgeons should limit the PCL drill guide by referring to the specific PSA for different surgical approaches.

## Graphical abstract

The permissive safe angle to avoid the posterior wall fracture of tibial tunnel in transtibial PCL reconstruction: a three‐dimensional simulation study.

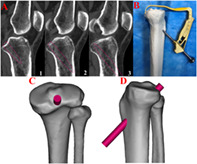

## Introduction

Both the posterior cruciate ligament (PCL) and the anterior cruciate ligament (ACL) are essential stabilizing structures of the knee joint.^1^, ^2^ However, as reported by previous studies, the surgical revision rate of the ACL reconstruction was 3%–5%, but the revision rate for PCL reconstruction was 26%–27%.^3^, ^4^ Therefore, the prognosis of PCL reconstruction was not encouraging compared with ACL reconstruction.^3^, ^4^, ^5^, ^6^, ^7^, ^8^ The killer turn created at the edge of the tibial tunnel has been regarded as one of the main reasons for the poor prognosis.^9^ The sharp graft angulation placed high compressive force on the graft at the tunnel margin of the proximal tibia, which would gradually abrade and stretch the PCL graft.^10^, ^11^, ^12^

Currently, two techniques are commonly used to minimize the killer turn effect, including the inlay technique or maximizing tibial tunnel angle technique.^13^, ^14^, ^15^, ^16^ As the inlay technique was associated with complex surgical procedures and high injury risks to the neurovascular structures in the popliteal fossa.^4^, ^17^ Therefore, the transtibial technique was more popular for PCL reconstruction.^4^, ^11^, ^18^ In transtibial PCL reconstruction, maximizing tibial tunnel angle could theoretically reduce the killer turn effect.^15^, ^19^ Nevertheless, some studies have shown that an excessive angle in transtibial PCL reconstruction may result in the posterior wall fracture of tibial tunnel.^15^, ^19^

Clinically, the posterior wall fracture of tibial tunnel increased potential risk of iatrogenic popliteal neurovascular injury.^20^ Although this complication rarely occurred, it might cause devastating results to patients.^21^, ^22^ In addition, the fracture of tibial tunnel theoretically affected the tendon‐bone healing of PCL grafts.^23^ Therefore, a safe angle of tibial tunnel was critical for PCL reconstruction. In order to avoid fractures on the tibial tunnel posterior wall while reducing the killer turn effect, several researchers have explored the maximum tibial tunnel angle of the transtibial PCL reconstruction based on the knee's CT image.^15^, ^19^ Unfortunately, these studies are limited to the sagittal plane, and the entry position of the tibial tunnel is located on the tibial crest. In clinical practice, the tibial tunnel entry position is commonly located anteromedial or anterolateral to the tibial crest. Because the anteromedial and anterolateral proximal tibia have different anatomic characteristics,^24^ the maximum angle of the tibial tunnel is theoretically varied when the location of the tibial tunnel approach changed in three‐dimensional (3D) space. Consequently, using two‐dimensional technique to evaluate the tibial tunnel angle might result in inaccurate outcomes which is limited to be used in the clinical practice. However, to date, few studies provided reference data for permissive safe angle (PSA) of tibial tunnel in transtibial PCL reconstruction based on the 3D knee model.

The 3D knee model simulation is a novel method which could provide an *in‐vitro* virtual technique to simulate the tibial tunnel.^25^ Previous studies have reported, the 3D knee model could be used in precise quantitative analysis and has excellent measurement accuracy and reliability.^26^ Based on the 3D knee model, the tibial tunnel could be monitored in real time, and the PSAs in different tibial tunnel approaches could be accurately measured and analyzed in 3D space. The purpose of this study was to: (i) establish the 3D knee model to simulate the transtibial PCL reconstruction; (ii) determine the PSAs in the transtibial PCL reconstruction in different tibial tunnel approaches based on the 3D knee model; (iii) explore the effects of patients' characteristics (sex, age and height) on important parameters of the tibial tunnel, such as the tibial tunnel height (TTH), tibial tunnel depth (TTD) and PSA. The hypothesis of this study was that the PSA varied with the anteromedial, tibial crest and anterolateral approaches for transtibial PCL reconstruction, and the specific PSA for different surgical approaches should be referred by surgeons to limit the angle of the PCL drill guide.

## Methods

### Sample Selection

The regional ethics committee of the institute approved this study (D2020‐29), and the computed tomography (CT) data with ultrahigh resolution of 90 knee joints from 2017 to 2020 were retrospectively reviewed. The inclusion criteria were as follows: (i) patients were 18–60 years of age; (ii) the CT images could be clearly identified the tibial attachment of the PCL; (iii) Kellgren–Lawrence grade less than 1. The images with: (i) dysplasia or deformities of the knee joint; (ii) fractures of the knee joint, any ligament knee injuries; and (iii) history of knee surgery were excluded in this study.

### 3D Reconstruction of Computed Tomography Imaging

All included patients underwent routine clinical knee CT performed on a 64‐multidetector‐row CT (SOMATOM Sensation, Siemens AG, Munich, Germany). Scanning parameters included a gantry rotation speed of 1.00 s/rotation, 0.625 mm collimation width × 12 detectors, a CT pitch factor of 0.90, and a field of view of 25–30 cm. The CT dose index (CTDI) volume was 20.9 mGy. The CT images were imported in Digital Imaging and Communications in Medicine (DICOM) format, and the axial computed tomography scan slices were segmented with Mimics (Version 21, Materialise, Leuven, Belgium) to generate the 3D knee models.

### Simulation of Transtibial Posterior Cruciate Ligament Reconstruction on 3D Knee Models

#### The Method to Determine the Tibial PCL Attachment Point

**Fig. 1:**
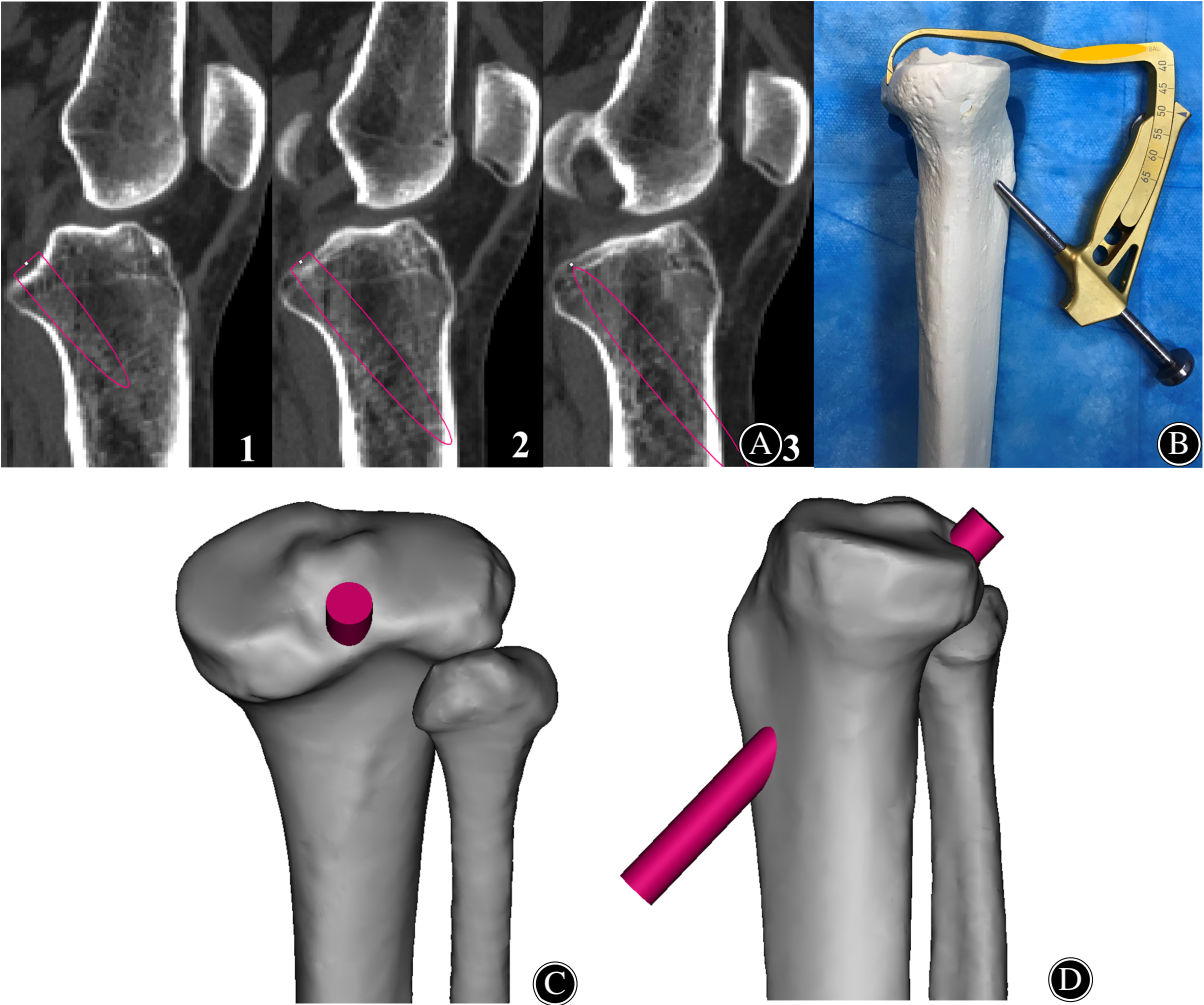
The tibial tunnel is simulated by the combined use of sagittal window and 3D window in Mimics software. (A) The PCL attachment location, the widest, clearest and most inclusive on the sagittal CT image of the tibia (Subfigure 1). The continuous sagittal scan images ensure that the simulated tunnel and the posterior tibial cortex are closed (Subfigure 1–3). (B) Posterior cruciate ligament guide system was position ed. on the proximal tibial cortex. And the tibial tunnel could be obtained based on the drill guide system. (C) Ensure that there is no breakage on the posterior cortex of the tibia on the posterior view of the 3D model of the knee joint. (D) Ensure that the entrance of the tibial tunnel is on the anterior 1/3 of the anteromedial tibia (T1) of the 3D model of the knee joint.

Based on the 3D simulation software, the tibial tunnel was simulated in the transtibial PCL reconstruction (Figure 1B). According to previous study,^15^ the tibial PCL attachment was located on the sagittal CT image using the widest and most clear attachment (Figure 1A). The PCL tibial attachment was regarded as the tibial tunnel exit point, and cylinders with a radius of 5 mm were built to simulate the tibial tunnels. The position of the cylinder was slightly adjusted on the 3D knee model to ensure that it was accurately showing the exit was at the tibial PCL attachment point (Figure 1C), which referred to the MRI measurement data obtained from a previous study of the tibial PCL attachment point.^27^

#### The Method to Simulate Different Tibial Tunnel Approaches

**Fig. 2:**
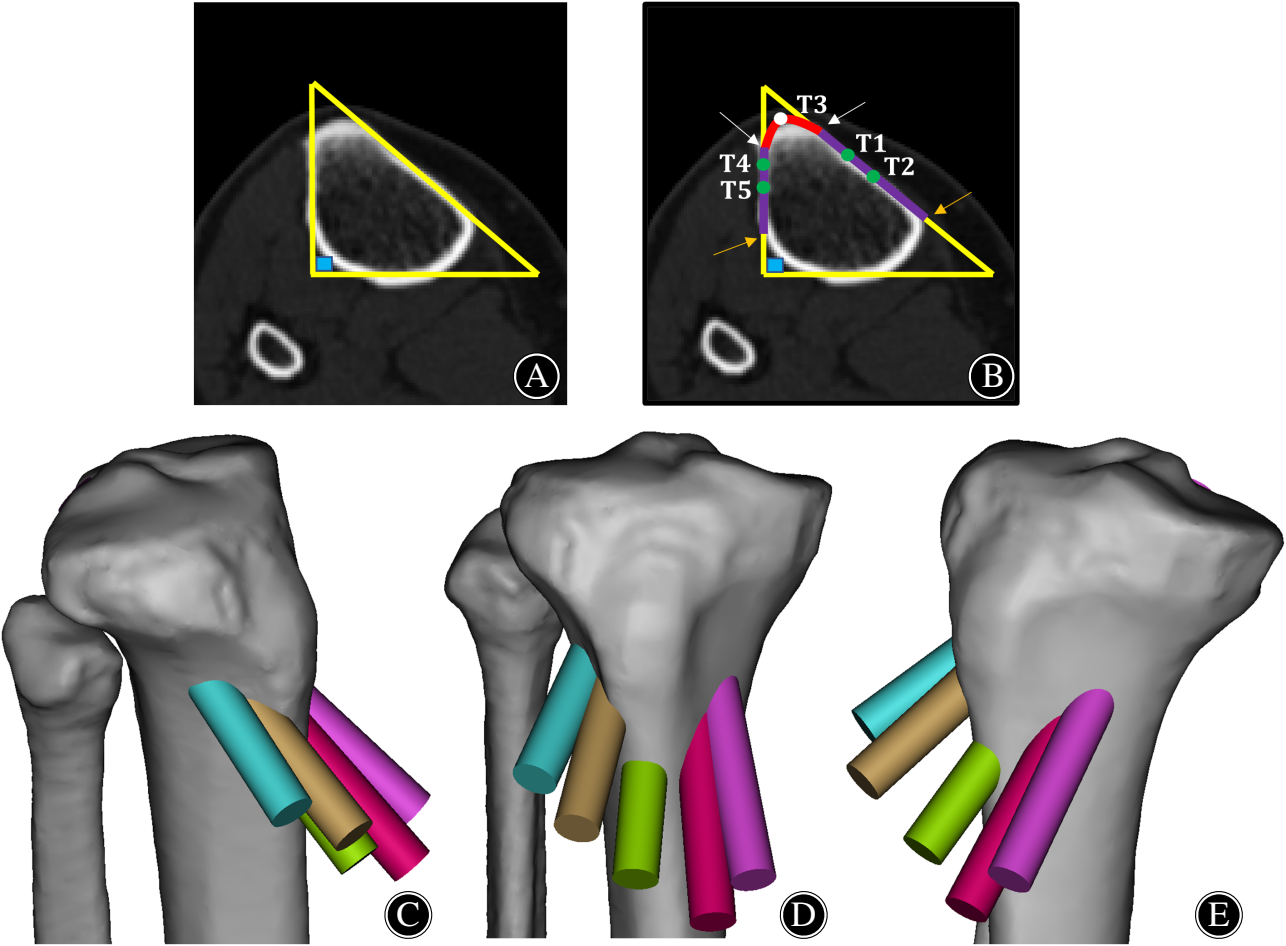
The tibial tunnels in different approaches. (A) On the cross‐section of the tibia, a right triangle is formed by the extension line of the anteromedial cortical edge of the tibia, the extension line of the anterolateral cortical edge of the tibia and the tangent line of the posterior cortex of the tibia. (B) Five different tunnel entrances are located on the cross‐section of the tibia based on Figure 2A. The junctions of the red line and the two purple lines (the white arrows) are the turning of the anteromedial cortex to the tibial crest and the turning of the anterolateral cortex to the tibial crest, respectively. The back ends of the two purple lines (the orange arrows) are the backward turning of the anteromedial tibial cortex and the backward turning of the anterolateral tibial cortex, respectively. The white dot is the center of the red arc, and the green dots are located at the anterior 1/3 and the midpoint of the two purple lines, respectively. (C) The anteromedial view of the 3D knee model with the five tibial tunnels. (D) The anterior view of the 3D knee model with five tibial tunnels. (E) Anterolateral view of the 3D knee model with five tibial tunnels.

When locating the entrance of the tibial tunnel, a right triangle was formed on the proximal tibial cross section as described by a study by Noyes *et al*.,^28^ in which the anteromedial tibial cortex has an oblique or triangular shape, whereas the anterolateral tibial cortex is almost perpendicular to the posterior margin of the tibia (Figure 2A). The midpoint of the tibial crest was located on the cross section, then the midpoint and the anterior 1/3 point of the parts of the anteromedial and anterolateral tibia cortex that overlapped with the triangle were located respectively (Figure 2B). Based on the above five points, the different tibial tunnel approaches were defined as the anterior 1/3 of the anteromedial tibia (T1), middle 1/2 of the anteromedial tibia (T2), the tibial crest (T3), anterior 1/3 of the anterolateral tibia (T4), middle 1/2 of the anterolateral tibia (T5).

Ensuring that the exit of the tibial tunnel was fixed, the simulated tibial tunnel was adjusted on the 3D window to place the entrance of the tunnel on the anterior 1/3 of the anteromedial tibia (T1), and the tunnel was closed to the posterior cortex of the tibia on the sagittal window (Figure 1). The other four different tibial tunnels were obtained in the same way (Figure 2). Meanwhile, none of the above five tunnels caused rupture of the posterior tibial cortex, which was confirmed by observing different perspectives of the knee model. The above steps ensured that the tibial tunnel of all different approaches was the most inclined and that the angle between the tibial tunnel and the tibial plateau was the largest. Then, the centerlines of each simulated tibial tunnel were built to simplify the measurement. The intersection of the centerline with the anterior tibial cortex was used as the entrance point of the tunnel, and the intersection of the centerline with the tibial plateau slope was used as the exit point of the tunnel.

#### The Method to Measure the PSA, TTH and TTD

**Fig 3:**
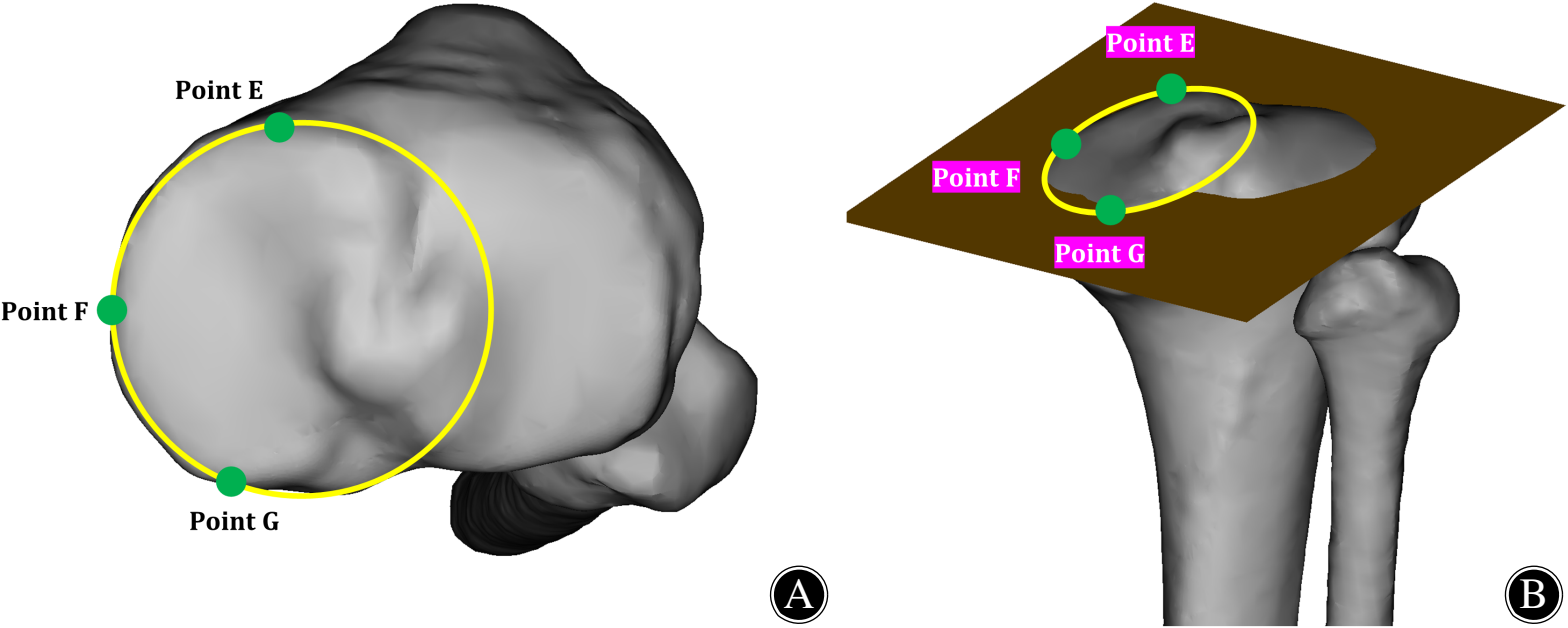
The establishment of the plane of the tibial plateau. (A) Establishment of three points of E, F and G on the tibial plateau of the 3D knee joint model by using the method of the best‐fit circle manually around the cortical edge of medial tibial plateau. Point E, the peak point of the anterior side of the medial tibial plateau. Point F, the most medial point of the tibial plateau. Point G, the peak point on the posterior side of the medial tibial plateau. (B) Anterior view of the 3D knee joint model. The plane of the tibial plateau was established by connecting three points in Figure 3A.

When measuring the PSA, TTD and TTH of each tibial tunnel, the referenced plane was the plane of medial tibial plateau,^13^, ^29^, ^30^ as the PCL femoral attachment was located medial condyle of femur and it was more easily visualized than the lateral tibial plateau during PCL reconstruction. As described by previous studies,^26^, ^31^ the plane of the medial tibial plateau was created using the method of the best‐fit circle, which was manually around the cortical edge of medial tibial plateau.^26^, ^31^ The three points at which the best‐fit circle tangent to the cortical edge of medial tibial plateau were defined as E (the peak point of the anterior side of the medial tibial plateau), F (the most medial point of the tibial plateau) and G (the peak point on the posterior side of the medial tibial plateau). The three points were connected to simulate the plane of the tibial plateau, which was defined as plane M (Figure 3).

**Figure 4:**
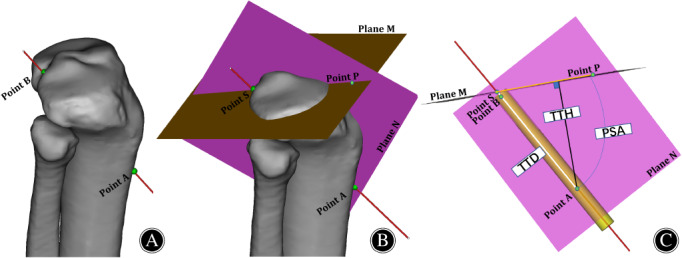
Measuring the anatomical data of the tibial tunnel in the 3D knee joint model. (A) Anterolateral view of the 3D knee joint model. Point A and point B represent the entrance and exit points of the tibial tunnel, respectively. (B) Plane N contains the centerline of the tibial tunnel and is perpendicular to plane M; Point P is any point on the intersection line of plane M and plane N; Point S is the intersection of the centerline of the tibial tunnel and plane M. (C) The perspective diagram of measuring the anatomical data of the tibial tunnel. The maximum safe angle of the tibial tunnel relative to the tibial plateau is the angle PSA; TTH is the distance from point A to plane M; TTD is the distance between the two points of AB.

Plane N containing the centerline of the tibial tunnel and perpendicular to plane M was constructed. Then, the entrance point of the tunnel was defined as point A, the exit point of the tunnel was defined as point B, the intersection of the centerline of the tibial tunnel with plane M was defined as point S, and any point was taken on the intersecting line of the plane M and N as point P (Figure 4). Then, Mimics software was used to accurately measure the PSA TTH and TTD in the 3D knee model.

### Measurements of the Tibial Tunnel on the 3D Knee Model

The anatomical features of the tibia were measured by two independent blinded observers. First, Observer 1 selected 10 knee joints randomly from all of the samples for pre‐experiment and data analysis. Then, the remaining 80 CT images were measured and analyzed after determining that the sample size was sufficient. One month later, all of the specimens were remeasured by Observer 2. If any disagreement existed between the observers, the third author would participate in the discussion until a consensus was reached. Intraclass correlation coefficient (ICC) was used to calculate the reliability of all outcomes.^32^ ICC < 0.40 was considered poor agreement; 0.4 < ICC < 0.75 was considered fair to good agreement; ICC > 0.75 was considered excellent agreement.^33^, ^34^

### Statistical Analysis

The *F* test (ANOVA: fixed effects, omnibus, one‐way) of G*Power software (version 3.1.9, Heinrich Heine University, Düsseldorf, Germany) was used to calculate the minimum sample size when the power (1‐β err prob) was 0.9. All data were processed by SPSS software (version 26.0, Chicago, IL, USA). Subgroup analysis was used to determine the correlation between the parameters and age, sex and height. The anatomical parameters of the tibial tunnel approaches, age and height cohort were analyzed by one‐way analysis of variance (ANOVA), while an independent *t*‐test was used between genders. The results are presented as the arithmetic mean ± standard deviation. *P* < 0.05 was considered statistically significant.

## Results

Based on G‐power analysis, the minimum sample size needed in this study was approximately 63. Consequently, it was sufficient for us to include 90 knee joints (37 right and 53 left; 34 male and 56 female) as the total number of samples. The average age of the patients was 37.2 ± 13.8 years (range, 16–60 years), and their average weight was 62.42 ± 11.11 kg. Intraclass correlation coefficients ranged from 0.84 to 0.95, which suggested an excellent intraindividual and interobserver agreements.

### Anatomical Data of Five Tibial Tunnels with Different Approaches

**Fig. 5:**
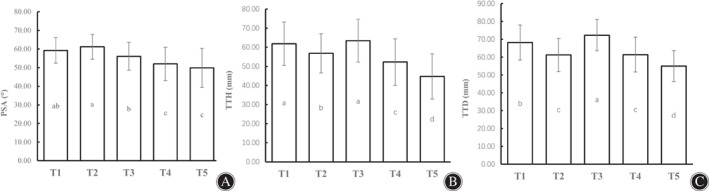
Anatomical data of five tibial tunnels with different approaches. (A) The PSA mean values of the tibial tunnel with 5 different approaches (T1–T5) were 59.27° ± 6.82°, 61.14° ± 6.69°, 56.12° ± 7.53°, 52.01° ± 8.89° and 49.90° ± 10.53°. (B) The mean TTHs of the tibial tunnel with 5 different approaches (T1–T5) were 61.86 ± 11.43 mm, 56.81 ± 10.34 mm, 63.47 ± 11.19 mm, 52.25 ± 12.21 mm and 44.79 ± 11.84 mm, respectively. (C) The mean TTDs of the tibial tunnel with 5 different approaches (T1–T5) were 68.21 ± 9.86 mm, 61.26 ± 9.29 mm, 72.36 ± 8.79 mm, 61.44 ± 9.80 mm and 55.04 ± 8.67 mm. Method of marked letters (a–e) is used to denote statistical differences. There is no significant difference between bars marked with a same letter (*P* > 0.05), while those without a same letter are significant (*P* < 0.05).

The mean PSAs of the T1–T5 approaches were 58.49° ± 6.82°, 61.14° ± 6.69°, 56.12° ± 7.53°, 52.01° ± 8.89° and 49.90° ± 10.53° (Figure 5). The mean PSA value in the anteromedial and anterolateral approaches of the tibial tunnel were significantly different (*P* < 0.05). However, there was no significant difference in the mean PSA value between the anteromedial T1 and T2 tibial tunnel approaches (*P* > 0.05), as well as between the anterolateral T4 and T5 tibial tunnel approaches.

Both the mean TTH and TTD of the anteromedial T1 and T2 tibial tunnel approaches and the anterolateral T4 and T5 tibial tunnel approaches decreased gradually with movement of the tunnel entrance from anterior to posterior *P* < 0.05). In addition, both the mean TTH and TTD of the anteromedial approaches were significantly increased compared with those of the corresponding anterolateral approaches (T1 vs. T4, T2 vs. T5).

### Subgroup Analysis in Terms of Sex, Age and Height

With respect to sex, there was no significant difference in the mean value of the PSA, but there were significant differences in the TTH and TTD between male and female (Table 1).

**TABLE 1:** Outcome parameters of gender groups

Parameter	Mean standard ± deviation		*F* value	*P* value
	Male (*n* = 34)	Female (*n* = 56)		
PSA 1 (°)	60.52 ± 6.36	58.51 ± 7.04	1.861	0.176
PSA 2 (°)	62.20 ± 6.35	60.50 ± 6.87	1.384	0.243
PSA 3 (°)	57.62 ± 6.73	55.22 ± 7.90	2.180	0.143
PSA 4 (°)	54.12 ± 8.71	50.72 ± 8.84	3.170	0.078
PSA 5 (°)	52.61 ± 10.79	48.25 ± 10.10	3.740	0.056
TTH 1 (mm)	67.27 ± 10.40	58.57 ± 10.83	14.058	0.000
TTH 2 (mm)	61.73 ± 9.72	53.82 ± 9.61	14.244	0.000
TTH 3 (mm)	68.19 ± 9.05	60.60 ± 11.45	10.811	0.001
TTH 4 (mm)	57.02 ± 11.40	49.35 ± 11.85	9.131	0.003
TTH 5 (mm)	48.64 ± 12.35	42.46 ± 10.98	6.099	0.015
TTD 1 (mm)	73.83 ± 8.78	64.80 ± 8.93	21.937	0.000
TTD 2 (mm)	65.95 ± 9.53	58.41 ± 7.96	16.308	0.000
TTD 3 (mm)	76.59 ± 8.15	69.79 ± 8.21	14.585	0.000
TTD 4 (mm)	64.46 ± 10.63	59.61 ± 8.87	5.432	0.022
TTD 5 (mm)	58.07 ± 9.39	53.20 ± 7.73	7.124	0.009

*Note*: PSA (1–5) the permissive safe angels between the tibial tunnels (TI–T5) and tibial plateau; TTH (1–5) the distance from the entry points of tibial tunnels (TI–T5) to the plane of the tibial plateau; TTD (1–5) the distance from the entrances to exit of the tibial tunnels (TI–T5).

With respect to age, the included patients were divided into a young group (18–30), a middle‐aged group (31–45) and an elderly group (46–60) (Table 2). The outcome parameters showed that the mean value of PSA of T4 in the middle‐age group was significantly lower than that in the young (*P* = 0.026), Both the TTH and TTD (T4 and T5) of the middle‐age and the elderly groups were significantly higher than that of the young group (*P* < 0.05).

**TABLE 2:** Outcome parameters of various age groups (Mean ± SD)

Age group (years)	Young (16–30), *n* = 35	Middle age (31–45), *n* = 28	Elderly (46–60), *n* = 27	*F* value		
				Young versus middle	Young versus elderly	Middle versus elderly
PSA 1 (°)	60.22 ± 7.07	57.85 ± 6.56	59.51 ± 6.79	1.869	0.158	0.856
PSA 2 (°)	61.72 ± 6.67	59.97 ± 6.70	61.60 ± 6.81	1.073	0.005	0.804
PSA 3 (°)	57.48 ± 8.04	54.34 ± 7.39	56.22 ± 6.85	2.537	0.425	0.950
PSA 4 (°)	54.55 ± 9.99	49.53 ± 8.24*	51.28 ± 7.34	4.563	2.037	0.686
PSA 5 (°)	52.65 ± 11.79	48.18 ± 10.29	48.11 ± 8.42	2.502	2.869	0.001
TTH 1 (mm)	63.44 ± 14.14	61.57 ± 9.29	60.10 ± 9.43	0.365	1.123	0.339
TTH 2 (mm)	58.20 ± 12.15	55.98 ± 9.42	55.85 ± 8.75	0.631	0.723	0.003
TTH 3 (mm)	66.13 ± 13.98	61.85 ± 8.16	61.68 ± 9.38	2.059	2.027	0.005
TTH 4 (mm)	56.41 ± 14.50	49.15 ± 8.81*	50.07 ± 10.77*	5.411	4.620	0.120
TTH 5 (mm)	50.14 ± 14.29	41.48 ± 8.93*	41.30 ± 8.17*	7.830	8.239	0.006
TTD 1 (mm)	69.44 ± 12.13	68.90 ± 7.34	65.90 ± 8.75	0.043	1.643	1.905
TTD 2 (mm)	62.79 ± 10.82	60.24 ± 8.88	60.32 ± 7.44	1.011	1.027	0.001
TTD 3 (mm)	73.95 ± 11.23	72.09 ± 5.71	70.58 ± 7.68	0.636	1.779	0.682
TTD 4 (mm)	64.80 ± 11.28	59.41 ± 7.59*	59.21 ± 8.82*	4.699	4.512	0.008
TTD 5 (mm)	58.68 ± 10.67	53.27 ± 6.01*	52.14 ± 6.42*	5.725	7.928	0.460

*Note*: PSA (1–5) the permissive safe angels between the tibial tunnels (TI–T5) and tibial plateau; TTH (1–5) the distance from the entry points of tibial tunnels (TI–T5) to the plane of the tibial plateau; TTD (1–5) the distance from the entrances to exit of the tibial tunnels (TI–T5). Compared to the young. *P < 0.05

With respect to height, the included patients were divided into three groups by height: (i) <1.60 m height group; (ii) 1.60–1.70 m height group; and (iii) >1.70 m height group). The results showed that no differences were found in the PSA among different height groups (*P* > 0.05). However, height affected the TTH and TTD (Table 3).

**TABLE 3:** Outcome parameters of various height groups (Mean ± SD)

Height group (m)				*F* value		
	(1) <1.60 m, *n* = 30	(2) 1.60–1.70 m, *n* = 40	(3) >1.70 m, *n* = 20	(1) versus (2)	(1) versus (3)	(2) versus (3)
PSA 1 (°)	58.00 ± 6.46	59.84 ± 7.52	60.04 ± 5.87	1.149	1.279	0.011
PSA 2 (°)	60.58 ± 6.49	61.28 ± 7.40	61.70 ± 5.70	0.170	0.393	0.050
PSA 3 (°)	54.79 ± 7.26	56.64 ± 8.33	57.10 ± 6.21	0.941	1.354	0.047
PSA 4 (°)	50.23 ± 8.75	52.38 ± 9.73	53.92 ± 7.09	0.908	2.470	0.396
PSA 5 (°)	47.41 ± 10.19	50.52 ± 11.41	52.39 ± 8.74	1.394	3.205	0.416
TTH 1 (mm)	57.25 ± 11.20	63.36 ± 11.74*	65.77 ± 9.11*	4.824	8.014	0.648
TTH 2 (mm)	53.25 ± 10.59	57.89 ± 10.13	59.97 ± 9.27*	3.462	5.318	0.590
TTH 3 (mm)	58.65 ± 9.70	64.61 ± 12.33*	68.41 ± 8.11*	4.786	13.812	1.559
TTH 4 (mm)	47.22 ± 10.95	52.81 ± 12.55*	58.67 ± 10.39*	3.778	13.651	3.241
TTH 5 (mm)	40.62 ± 10.44	45.49 ± 12.70	49.67 ± 10.28*	2.917	9.110	1.630
TTD 1 (mm)	63.76 ± 9.30	69.19 ± 10.19*	72.94 ± 7.31*	5.231	13.780	2.513
TTD 2 (mm)	58.06 ± 8.87	62.28 ± 9.07*	64.00 ± 9.45*	3.778	5.114	0.468
TTD 3 (mm)	68.25 ± 6.75	73.12 ± 9.34*	77.01 ± 7.93*	5.861	17.622	2.556
TTD 4 (mm)	57.90 ± 8.00	61.79 ± 9.90	66.06 ± 10.44*	3.115	9.773	2.387
TTD 5 (mm)	51.31 ± 7.57	55.82 ± 8.67*	59.05 ± 8.40*	5.159	11.491	1.889

*Note*: PSA (1–5) the permissive safe angels between the tibial tunnels (TI–T5) and tibial plateau; TTH (1–5) the distance from the entry points of tibial tunnels (TI–T5) to the plane of the tibial plateau; TTD (1–5) the distance from the entrances to exit of the tibial tunnel (TI–T5). Compared to 1. *P < 0.05.

## Discussion

### The Main Findings of this Study

The most important finding of this study was that the PSA significantly varied with different approaches from the anteromedial, tibial crest to anterolateral tibial tunnels, and the mean PSAs of five tibial tunnels (T1–T5) relative to the tibial plateau were 58°, 61°, 56°, 52°and 50°. In order to optimize the position of tibial tunnel, surgeons should limit the PCL drill guide angle using a specific PSA to minimize the killer turn and avoid posterior wall fracture during transtibial PCL reconstruction.

### The Importance of the PSA

Recurrent posterior relaxation is one of the most common complications after PCL reconstruction.^10^ Previous studies have revealed that the sharp graft angulation between the graft and the tibial plateau caused a high compressive force to the graft in transtibial PCL reconstruction, which was named as killer turn.^4^, ^11^, ^12^, ^35^, ^36^ Several studies have found that the killer turn can be reduced by increasing the angle of the tibial tunnel relative to the tibial plateau.^12^, ^15^ Nevertheless, an excessive angle could cause fractures to the posterior tibial cortex, which might result in iatrogenic injuries to the neurovascular bundles in the posterior popliteal fossa.^8^, ^16^, ^23^, ^36^ Consequently, the PSA should be determined in the transtibial PCL reconstruction.

To determine the PSA of the tibial tunnel, Lee *et al*.^19^ performed a cadaveric study using 10 fresh tibias, and they found that the maximum safe angle of the tibial drill guide was 52° based on CT data. Teng *et al*.^15^ used CT images to simulate transtibial PCL reconstruction, and the results showed that the maximum angle of the tibial tunnel was 48.2°. However, a major limitation regarding the above studies was that when PCL tibial tunnel was simulated on 2D sagittal CT images, the tunnel approach would be located on the tibial crest. In fact, surgeons usually used the anteromedial or anterolateral approach for transtibial PCL reconstruction. To date, no consensus has been reached regarding the safe angle for tibial tunnel.

### The PSA in Different Tibial Tunnels

**Fig. 6:**
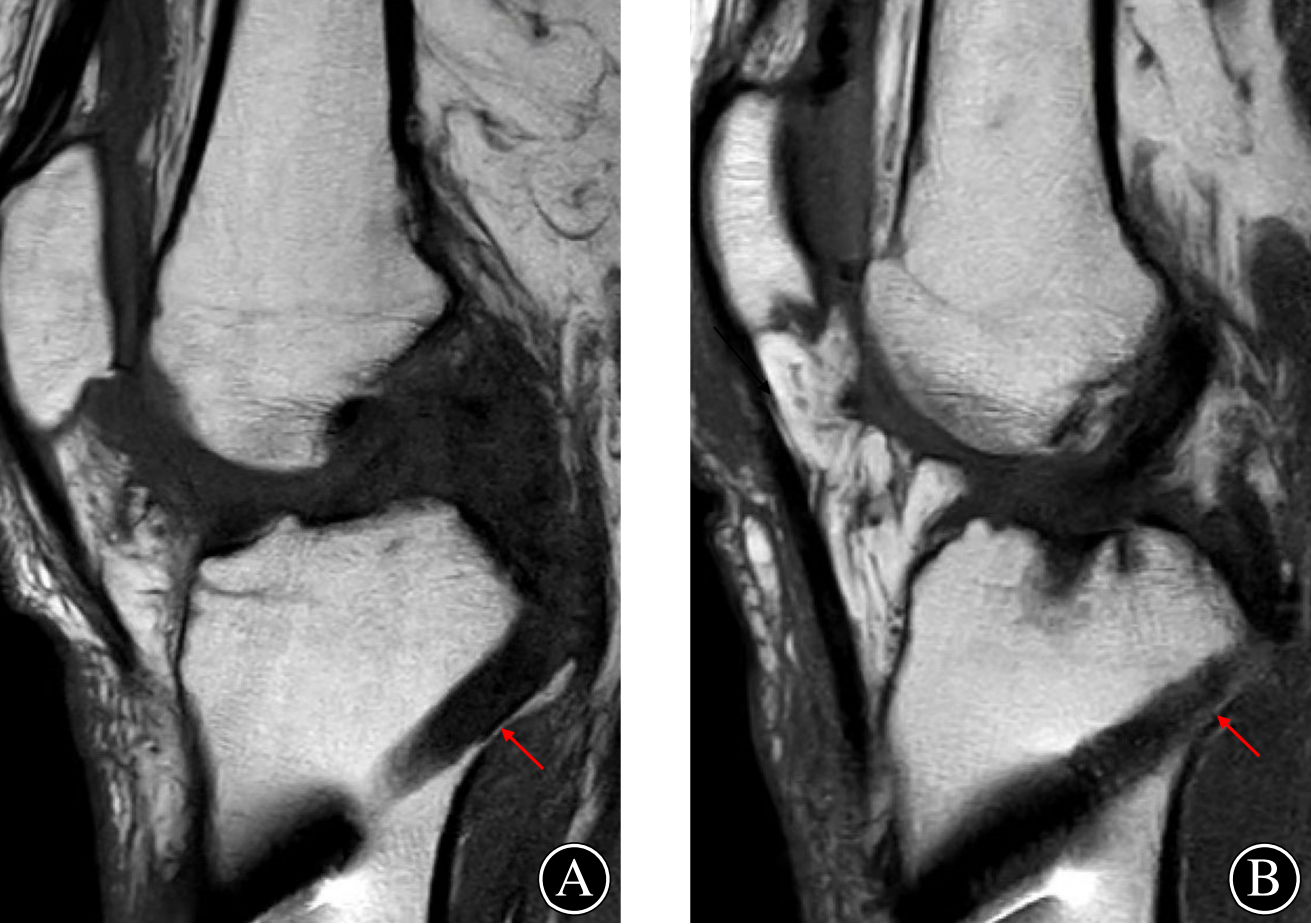
Two cases of PCL tibial tunnels on MRI images: red arrows showed that the posterior wall of tibial tunnel was very close to the posterior tibial cortex.

In this study, the surgical procedure of the transtibial PCL reconstruction was simulated based on the 3D knee models. The PSA was defined as the permissive safe angle between the tibial tunnel and tibial plateau, which was consistent with the angle of the PCL drilling guide. Our results showed that the mean value of the PSA significantly varied in the different approaches. The mean values of PSA were approximately 58° and 61° for the anteromedial T1 and T2 approaches, 56° for the tibial crest T3 approach, and 52° and 50° for the anterolateral T4 and T5 approaches. These values were of great importance for surgeons to obtain an optimal position of PCL tibial tunnel. To minimize the killer turn effect during PCL reconstruction, surgeons tend to maximize the tibial tunnel angle, making the posterior wall of tibial tunnel very close to the posterior tibial cortex (Figure 6). However, an increased tibial tunnel angle had higher risks in terms of the tibial tunnel fracture and popliteal neurovascular bundle. In our study, we provided essential reference values for surgeons to set a maximum and safe angle for the PCL drilling guide.

In addition, surgeons should note that the PSA was different when they used an anteromedial or anterolateral approach for PCL reconstruction. Currently, there is no consensus on the superior approach between anteromedial and anterolateral approach in PCL reconstruction. Kim *et al*.^37^ performed a finite element and cadaveric study to investigate the effect of three different tibial tunnels (medial, central and lateral) on the stress concentration around the killer turn in PCL reconstruction. They found that the medial approach had the highest stresses, and the lateral approach had the lowest stresses to PCL grafts. A biomechanical study also provided similar conclusions for anteromedial and anterolateral approaches. They found that the anterolateral approach showed less graft angulation at the graft tunnel margin.^10^ However, Ahn *et al*.^24^ compared the fixation strength between anteromedial and anterolateral tunnels in PCL reconstruction, and found that the anterolateral approach was associated with a lower failure load compared with the anteromedial approach. At present, most surgeons choose the surgical approach of the PCL tibial tunnel based on their preferences. However, the recommended entry points of guide pin on the anteromedial or anterolateral cortex of the proximal tibia are still lacking.

This is the first study to divide the entry points of guide pin into five portals: anterior 1/3 of the anteromedial tibia, middle 1/2 of the anteromedial tibia, the tibial crest, anterior 1/3 of the anterolateral tibia and middle 1/2 of the anterolateral tibia. Based on our findings, the mean PSA was approximately 6° lager in the anterior 1/2 of the anteromedial approach than the anterior 1/2 of the anterolateral approach, and 11° lager in the anteromedial middle 1/2 approach than the anterolateral middle 1/2 approach. This difference should be kept in mind when surgeons used different surgical approaches for PCL reconstruction. If an excessive angle was used for PCL tibial tunnel, that might result in fracture to the posterior wall of PCL tibial tunnel.

### TTD and TTH in Different Tibial Tunnels

The reference value of the TTD cannot be ignored when performing transtibial PCL reconstruction. If the reamer is drilled too deep, it may cause iatrogenic injury to the posterior nerves and vessels.^22^, ^36^ The major finding of our study was that the TTD varied greatly depending on the different tunnel approaches. And the anteromedial TTD was significantly longer than the anterolateral. Therefore, the depth of the reamer should be limited according to the different tunnel approaches. Our findings suggest that the permissive safe depths of the T1–T5 approaches are 68.21, 61.26, 72.36, 61.44 and 55.04 mm.

When determining the location of the entrance to the tibial tunnel, the TTH is a key parameter that can assist in precise positioning. In the present study, the mean permissive heights of the T1–T5 approaches were 61.86, 56.81, 63.47, 52.25 and 44.79 mm. It should be noted that the TTH of T1–T2 and T4–T5 decreased gradually and the anteromedial TTH was significantly higher than the anterolateral. Taken together, these results suggest that the TTH outcomes should be combined with the PSA to provide an easy and more accurate and complementary method to locate the tunnel entrance.

### Subgroup Analysis

Subgroup analysis showed that sex, age and height affected TTH and TTD to different degrees. Therefore, when performing transtibial PCL reconstruction surgery, individual differences should be noted to limit the length of the guide wire and reamer to avoid iatrogenic injuries to nerves and vessels in the popliteal fossa and to use an appropriate TTH to locate the entrance of the tibial tunnel in different approaches. In other words, when performing transtibial PCL reconstruction, surgeons should not only consider the mean value of the PSA but should also pay attention to the individual differences between TTH and TTD.

### Limitations

This research has the following limitations: (i) this study is a theoretical study under ideal conditions, which might have somewhat different outcomes with real clinical practice. The conclusion should be further verified by basic biomechanical experiments and clinical study to explore the feasibility of PSA used in different approaches of the transtibial PCL reconstruction; (ii) T3 are rarely used by surgeons during transtibial tunnel PCL reconstruction but does not rule out the possibility of using them if necessary. In addition, T3 can make this study more continuous to observe the changes of the relevant parameters of the tibial tunnel with the change of tunnel position; (iii) we relied on CT sagittal images to determine the tibial attachment site of the PCL. This method does involve potential error, which may have some deviation from the real original position, but based on a previous study of anatomical data of the PCL attachment point,^27^, ^38^ a small adjustment was made in the 3D window to reduce the deviation; and (iv) there are some other aspects on how to avoid fractures of the PCL tibial tunnel, for instance, the tunnel drilling techniques and the drilling speed. PSA should not be regarded as the only aspect to avoid fractures of the tibial tunnel and surgeons should take all factors into account during the transtibial PCL reconstruction.

### Conclusions

The PSA was different with the anteromedial, tibial crest and anterolateral approaches in transtibial PCL reconstruction. To obtain an optimal position of the tibial tunnel which could minimize the killer turn and avoid posterior wall fracture, surgeons should use a specific angle to limit the PCL drill guide while using different surgical approaches.

## Funding Information

This work was supported by the Gansu Natural Science Foundation Youth Project (21JR1RA154); National Natural Science Foundation of China (82060413); Innovation Fund for Universities in Gansu Province (2020B‐029); Lanzhou Science and Technology Plan Project (2021‐1‐106); Cuiying Scientific Training Program for Undergraduates of Lanzhou University Second Hospital (CYXZ2021‐17/CYXZ2021‐25).

## Conflicts of Interest

All the authors declare that there is no conflict of interest.

## Informed Consent

The data was derived from Computed Tomography images. The authors declare that there is no identifying information in any case and no reused image from prior publications.

## Authorship

We declare that each author (Yuanjun Teng, Gengxin Jia, Lijun Da, Bo Peng, Zhongcheng Liu, Hua Han, Meng Wu, Yayi Xia) has participated the following work for this manuscript.

1. Substantial contributions to the conception or design of the work; or the acquisition, analysis, or interpretation of data for the work;
2. Drafting the work or revising it critically for important intellectual content;
3. Final approval of the version to be published;
4. Agreement to be accountable for all aspects of the work in ensuring that questions related to the accuracy or integrity of any part of the work are appropriately investigated and resolved.

## Ethical Approval

Certificate of Institutional Review Board (IRB) Approval.

E‐Our work has been approved by medical ethics committee of Lanzhou University Second Hospital (D2020‐29).
